# Prevalence and associated factors of vernal keratoconjunctivitis among children in Gondar city, Northwest Ethiopia

**DOI:** 10.1186/s12886-016-0345-7

**Published:** 2016-09-29

**Authors:** Dereje Hayilu, Kbrom Legesse, Natinael Lakachew, Mulusew Asferaw

**Affiliations:** 1 Department of Optometry, School of Medicine, College of Medicine and Health Sciences, University of Gondar, Gondar, Ethiopia; 2 Department of Ophthalmology, School of Medicine, College of Medicine and Health Sciences, University of Gondar, Gondar, Ethiopia

**Keywords:** Vernal keratoconjunctivitis, Children, Gondar, Ethiopia

## Abstract

**Background:**

Vernal keratoconjunctivitis (VKC) is a common cause of ocular morbidity in children in warm dry climates such as Sub–Saharan Africa and accounts for about 3 % of serious ophthalmic cases in tropical countries. The purpose of this study was to assess the prevalence and associated factors of vernal keratoconjunctivitis among children living in Gondar City, Ethiopia.

**Methods:**

A Cross Sectional Design study was carried out in 737 children under the age of 18 years in Gondar City from April to May 2015. Basic ophthalmic examination was performed using a 3x magnifying loop and torch light and a pretested and structured questionnaire was completed. The association between vernal keratoconjunctivitis and factors such as socio-economic, demographic, and environmental status, and history of allergic disease in affected children and their family members was examined using logistic regression multivariate analysis.

**Results:**

The prevalence of vernal keratoconjunctivitis was 5.8 % (95 % CI: 4.14, 7.53) (43/737) and mixed type VKC was the most frequent form which was found in 35 out of 43 cases (81.4 %). The following were positively associated with vernal keratoconjunctivitis: use of kerosene/firewood for cooking (AOR = 6.25 (95 % CI: 1.61, 25)), child dust exposure (AOR = 10.0 (95 % CI: 4.16, 20.0)), child history of non-ocular allergic diseases (AOR = 4.0 (95 % CI: 1.92, 8.33)), family history of non-ocular allergic diseases (AOR = 3.57(95 % CI: 1.39, 9.09).

**Conclusion:**

There is a high prevalence of vernal keratoconjunctivitis in this region. The use of kerosene/firewood for cooking, child dust exposure, and non-ocular allergic disease in the child or their family were statistically significant risk factors for vernal keratoconjunctivitis.

## Background

Vernal keratoconjunctivitis (VKC) is a chronic, recurrent bilateral inflammation of conjunctiva and cornea that tends to occur in children and young adults. It presents with intense itching, swollen eyelid, tearing, red eye, foreign body sensation, mucous discharge and photophobia. The most common signs are lid edema, chemosis, tarsal papillae, Horner-Trantas dots, limbal infiltrates (limbitis), giant papillae and corneal epitheliopathy. Based on the above clinical presentation, VKC is classified into: palpebral, limbal and mixed VKC [1, 2].

VKC has a global distribution with a widely varying incidence. It is less common in Northern Europe and North America but relatively common in warm dry climates such as the Mediterranean countries, Central and South America, Sub – Saharan Africa and the Middle East. It accounts for about 1 % of eye disease in most parts of the world and 3 % of serious ophthalmic disease in tropical countries [3, 4].

Males are affected twice as often as females with a peak incidence between the ages of 11 and 13 years old, although 10 % of VKC patients are older than 20 years old at the time of onset. A large number of patients with VKC have symptoms that are exacerbated in the spring, possibly due to the increase in pollen count [5–7].

Vernal Keratoconjunctivitis is an allergic response that is difficult to prevent, but can be managed. Preventive measures include regular hand and face washing, staying out of the sun, keeping away from dust and smoke, and avoiding touching or rubbing of the eyes. Medical treatment is also important in controlling the disease [8–10].

Previous epidemiological studies of VKC in Ethiopia have been undertaken in a different environmental setting and concerned a different ethnic group compared to that found in Gondar [11–13] and there is no community based report on the prevalence and risk factors associated with VKC in North West Ethiopia. Our study aimed to assess the prevalence of VKC and the factors associated with VKC in children in order to implement preventive and intervention programs in this region.

## Methods

### Study design, setting and sampling

A population based cross sectional survey of households with children under the age of 18 years old was conducted in Gondar City, northwest Ethiopia, from April – May, 2015. Gondar has an altitude of 2,200 m above sea level with warm and wet weather. It has 10 sub-cities and 21 Kebeles hosting approximately 53, 725 households and 100, 984 children under 19 years of old. A Kebele is defined as the smallest administrative unit consisting of at least 2000 households [14].

A sample size of 744 participants was determined based on power calculations using data from a similar study from Rwanda [15], assuming 95 % CI, 80 % power, ratio of control to case 3:1, 42.% of cases exposed to dust, odds ratio 2.1 for dust exposure variable, two sampling stage and a 10 % non- response rate. In the study, 737 study participants were recruited and completed a questionnaire along with a basic ophthalmic examination. This corresponds to a 99.06 % response rate.

The study participants were selected using a multistage sampling method. Initially, four Kebeles from a total of 21 Kebeles were selected randomly. The sample for each Kebele was proportionally allocated according to the size. The households were randomly selected using a systematic random sampling interval of 17 with the assumption that there was at least one child in each household. When there was no child in the selected household, the next house was considered. If there was more than one child, only one child was randomly selected. Children between the ages of 2 and 18 years old who had been resident for the previous 6 months in Gondar were included in the study. Participants with other forms of ocular allergy, ocular infection, recent ocular trauma or surgery during the survey were excluded from the study.

The study was conducted in accordance with the Declaration of Helsinki and approved by the University of Gondar Ethical Review Board. In accordance with the Ethiopian National Research Ethics Review Guideline, assent was obtained from all children and verbal informed consent was obtained from the parents, next-of-kin or guardians using an information sheet in the local language “Amharic”. Those children who had VKC were referred to the University of Gondar tertiary eye hospital for detail examination and management.

### Definition of Vernal Keratoconjunctivitis

Vernal Keratoconjunctivitis is defined as the presence of tarsal and or limbal papillae ≥ 1 mm diameter with itching sensation and at least one of the following symptoms in the last 6 months: photophobia, sticky mucus discharge, redness, tearing and foreign body sensation.

*Palpebral* Vernal Keratoconjunctivitis is defined as the presence of papillae > 1 mm on the tarsal conjunctiva without limbal involvement with itching sensation and at least one of: photophobia, sticky mucus discharge, redness, tearing and foreign body sensation in the last 6 months.

*Limbal* Vernal Keratoconjunctivitis is defined as at least one of the following limbal findings: thickening, broadening, opacification, Horner-Trantas dots with itching sensation and at least one of the following symptoms in the last 6 months: photophobia, sticky mucus discharge, redness, tearing and foreign body sensation.

Cases were classified as Mixed Vernal Keratoconjunctivitis if they had the features of both limbal and tarsal VKC.

### Data collection and ophthalmic examination

One or two members of the pre-trained research team interviewed all children aged 15 years and above and the parents or guardians of children under the age of 15 years using a pre- tested and structured questionnaire in the Amharic language. Consistency was checked by back translation to English. A supervisor collected and reviewed the completed questionnaires daily. The questionnaire sought socio-demographic variables, self-reported history of non-ocular allergic diseases such as asthma, atopic dermatitis and rhinitis, symptoms related to VKC, and risk factors. Basic ophthalmic examination of the anterior segment was conducted inside the house using torch light and 3x magnifying loop by an experienced optometrist and ophthalmologist. Examination findings were recorded in English..

### Statistical analysis

The data were entered into EPI INFO 3.5.1 and SPSS version 20 was used for analysis. Frequencies and cross tabulation were used to check consistency. Bivariate logistic regression was used to determine the association between VKC and independent variables indicating the crude odds ratio. Crude odds ratio was calculated to show the strength of association between the outcome and single independent variables. Variables with *P*-value less than 0.2 at bivariate logistic regression were included in a multivariate logistic regression model to determine VKC risk factors adjusted for potential confounders. Adjusted odds ratio, 95 % CI and two sided *p*-value were calculated. A *p*-value less than 0.05 was considered statistically significant.

## Results

A total of 737 study participants, of which 376 (51 %) were male, were included in the study. The mean age (+ SD) of study participants was 8.7 years (+3.9) and 301 children (40.8 %) were in the age range of 6–10 years. More than half of the children (59.7 %) were primary school students. Of the total household heads, 244 (33.1 %) attended secondary school (Table 1).

**Table 1:** Socio-demographic characteristics of study participants among children living in Gondar city, Northwest Ethiopia, 2015

Variables	Frequency	Percent
Age category		
2-5	193	26.2
6-10	301	40.8
11-18	243	33.0
Sex		
Male	376	51
Female	361	49
Child education status		
preschool	249	33.8
primary	440	59.7
secondary	48	6.5
House hold education status		
Unable to write and read	94	12.8
Primary school	238	32.3
Secondary school	244	33.1
College/university	161	21.8

**Fig. 1:**
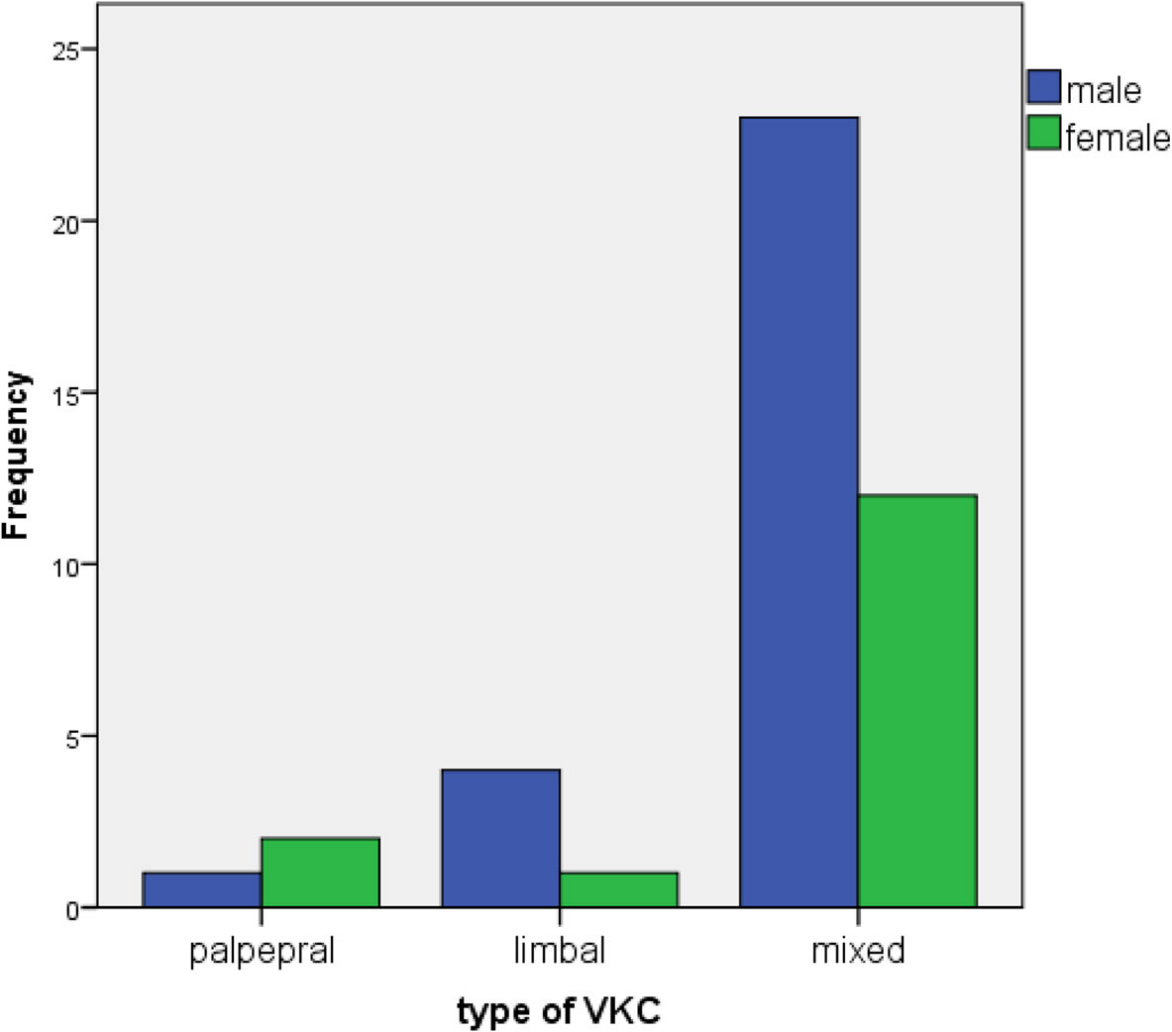
Sex and Type of vernal keratonjunctivitis in children living in Gondar City, Northwest Ethiopia, 2015

VKC was found in 43 out of 737 children giving a prevalence of 5.8 % (95 % CI: 4.14, 7.53). Twenty eight (65 %) of the affected children were male. Among the children with VKC, symptoms of redness and thick discharge were reported in 37 (86 %) and 27 (62.8 %) respectively. The signs and symptoms of VKC were exacerbated during the spring season in 30 out of 43 children with VKC (69.8 %). Thirty nine (90.7 %) children with VKC had bilateral papillary reaction/cobblestone on the tarsal conjunctiva. No patients had corneal ulcer (Table 2). Thirty five out of the 43 affected children (81 %) had Mixed VKC (Fig. 1).

**Table 2:** Magnitude and clinical features of vernal keratoconjunctivitis of study population among children living in Gondar city, Northwest Ethiopia, 2015

Variables	Frequency	Percent
VKC (*n* = 737)		
Yes	43	5.8
No	694	94.2
Symptoms(*n* = 43)		
Itching	43	100
Redness	37	86
Photophobia	23	53.5
Tearing	24	55.8
Thick discharge	27	62.8
Foreign body sensation	25	58.1
Exacerbation season for VKC (*n* = 43)		
Summer	6	13.9
Spring	30	69.8
Not specified	7	16.3
Sign (*n* = 43)		
Papillary reaction/cobblestone	39	90.7
Mucoid discharge	23	53.5
Hyperemia	25	58.1
Conjunctiva swelling	11	25.6
Limbal opacifications	26	60.5
Limbal thickening	28	65.1
Limbal widening	30	69.8
Horner trantas dot	12	27.9
Superior corneal pannus	9	20.9
Superior punctuate keratitis	2	4.7
Corneal ulcer	--	--

In bivariate analysis, house floor made up of soil, the use of kerosene/firewood for cooking, child dust exposure, child history of non-ocular allergic diseases, family history of allergic eye diseases and family history of non-ocular allergic diseases were statistically associated with VKC. In multivariate logistic regression analysis, the use of kerosene/firewood for cooking, child dust exposure, child history of non-ocular allergic diseases and family history of non-ocular allergic diseases were independently associated with VKC.

This study showed that those exposed to kerosene/firewood for cooking were 6.25 times more likely to develop VKC compared to those using electric power for cooking (AOR = 6.25 (95 % CI: 1.61, 25)). Children exposed to dust were 10 times more likely to develop VKC than non-exposed (AOR = 10.0 (95 % CI: 4.16, 20.0)). Children with non-ocular allergic disease were 4 times more likely to develop VKC compared to those without non-ocular allergic diseases (AOR = 4.0 (95 % CI: 1.92, 8.33)). The children with family history of non-ocular allergic diseases were also 3.57 times more likely to have VKC compared to those without a family history of non-ocular allergic diseases (AOR = 3.57(95 % CI: 1.39, 9.09) as shown in Table 3.

**Table 3:** Factors associated with vernal keratoconjunctivitis of study population among children living in Gondar city, Northwest Ethiopia, 2015

Variables	VKC			
	Yes	No	COR (95 % CI)	AOR (95 % CI)
Age category in years				
2-5	8	185	1.00	
6-10	22	279	1.82 (0.79, 4.18)	
11-18	13	230	1.30 (0.53, 3.22)	
Sex				
Male	28	348	1.86 (0.97, 3.54)	
Female	15	346	1.00	
Child educational status				
Preschool	31	457	1.34 (0.68, 2.67)	
Primary/secondary school	12	237	1.00	
House hold educational status				
Unable to write and read	6	88	1.76 (0.55, 5.63)	
Primary school	16	222	1.86 (0.71, 4.86)	
Secondary school	15	229	1.69 (0.64, 4.46)	
College/university	6	155	1.00	
Number of rooms in the house				
1-2	11	239	1.00	
3 and above	32	455	0.65 (0.32, 1.32)	
House floor condition				
Ceramics/cemented	4	272	1.00	
Earth/soil	39	422	6.29 (2.22, 17.79)	
Sleeping material				
Foam	33	525	1.06 (0.51, 2.2)	
Cotton/hay	10	169	1.00	
Cooking room condition				
Separated room	18	330	1.00	
Within living room	10	217	0.84 (0.38, 1.86)	
Open field	15	147	1.87 (0.92, 3.81)	
Cooking material				
Firewood/kerosene	34	435	1.00	1.00
Electric power	9	259	0.44 (0.94, 0.21)	0.16 (0.62, 0.04)*
Distance of house from main road				
Up to 500 m	29	361	1.9 (0.99, 3.68)	
500 m and above	14	333	1.00	
Dust exposure				
No	8	508	1.00	1.00
Yes	35	186	11.95 (5.44, 26.2)	10.0 (4.16, 20.0)**
Child non-ocular allergic history				
No	26	627	1.00	1.00
Yes	17	67	6.12 (3.16, 11.85)	4 (1.92, 8.33)**
Family ocular allergic history				
No	27	626	1.00	
Yes	16	68	5.45 (2.8, 10.63)	
Family non-ocular allergic history				
No	28	652	1.00	1.00
Yes	15	42	8.32 (4.13, 16.76)	3.57 (1.39, 9.09)**

P-value *<0.05 **<0.001

## Discussion

This study indicates that VKC is a significant public health problem that calls for preventive and intervention measures. We found a prevalence of VKC of 5.8 % (43/737 95 % CI: 4.14, 7.53). This is consistent with a similar study conducted in Butajira, Ethiopia and in rural Haryana, India [11, 16]. These studies have suggested that endocrine, neurogenic, environmental and socio-economic factors which may vary with ethnicity and geographic location have an important impact on the prevalence of VKC. We found a higher prevalence of VKC higher than in the study conducted in rural south-eastern Nigerian community school children [17] and have demonstrated an association between VKC and a number of socioeconomic factors that may explain the high prevalence in our study.

As in previous studies, particularly from Europe and Asia, we found a male predominance in all types of VKC. As in previous studies from Africa and Asia Mixed type VKC was the predominant type [2, 3, 15].

The most commonly affected age group in our study was 6–10 years of age 22/43(51.12 %). This is in accordance with a study from Uganda which found the commonest presentation with VKC to be between 5 and 9 years of age [18]. This Ugandan study proposed that exposure to ultraviolet light and wind may be important with wind increasing exposure to allergens in dust and pollen. Children are particularly at risk because they prefer to spend much of their time outdoors in the first decade of life.

VKC showed seasonal variation in our study. The symptoms become more prominent during dry and hot spring season in more than half of the children with VKC. This is in agreement with a study conducted in Nigeria which reported a perennial presentation of VKC with seasonal variation [19] with a high chance of increased environmental allergens during the hot and dry spring season.

Use of kerosene/firewood for cooking, dust exposure, children with non-ocular allergic diseases and family history of non-ocular allergic disease were associated with increased risk of vernal keratoconjunctivitis. This association with the use of kerosene/firewood for cooking could be due to increased exposure to smoke, dust and heat. Similar results were reported from an eye clinic case-control study reported from Nigeria [20].

A case–control study conducted in Rwanda showed that in severe VKC, exposure to dust is the main risk factor. It is believed that this is caused by conjunctival hyper-reactivity when nonspecific stimuli come in contact with the conjunctival mucosa, although the mechanism for this is not understood.

The association we found between non-ocular allergic diseases and VKC is in line with previous studies from India, Thailand, Italy and Sweden [21–24]. This is believed to be due to common features in the immunopathology of asthma, bronchitis, eczema and hay fever including fixation of IgE molecules on the surface of mast cells and release of mediators such as histamine and prostaglandins which mediate a type one immune reaction [1, 2].

This study found an association between family history of non-ocular allergic disease, such as asthma, atopic rhinitis, and eczema and VKC. There are conflicting reports in the literature about this association with one study showing a high association while another study conducted by the same author indicated that VKC is commonly not associated with a family history of atopic diseases [7, 25].

This study has some important limitations: Much of the data is self-reported and subject to recall bias from the subjects. Another limitation of the study is that we may not have considered other, as yet unknown, variables and we did not undertake any laboratory investigations of our subjects..

## Conclusion

There is a high prevalence of vernal keratoconjunctivitis In Gondar City in North West Ethiopia. The use of kerosene/firewood for cooking, dust exposure, and the presence of non-ocular allergic disease in the child or family members of the child, are statistically significant risk factors for vernal keratoconjunctivitis.
